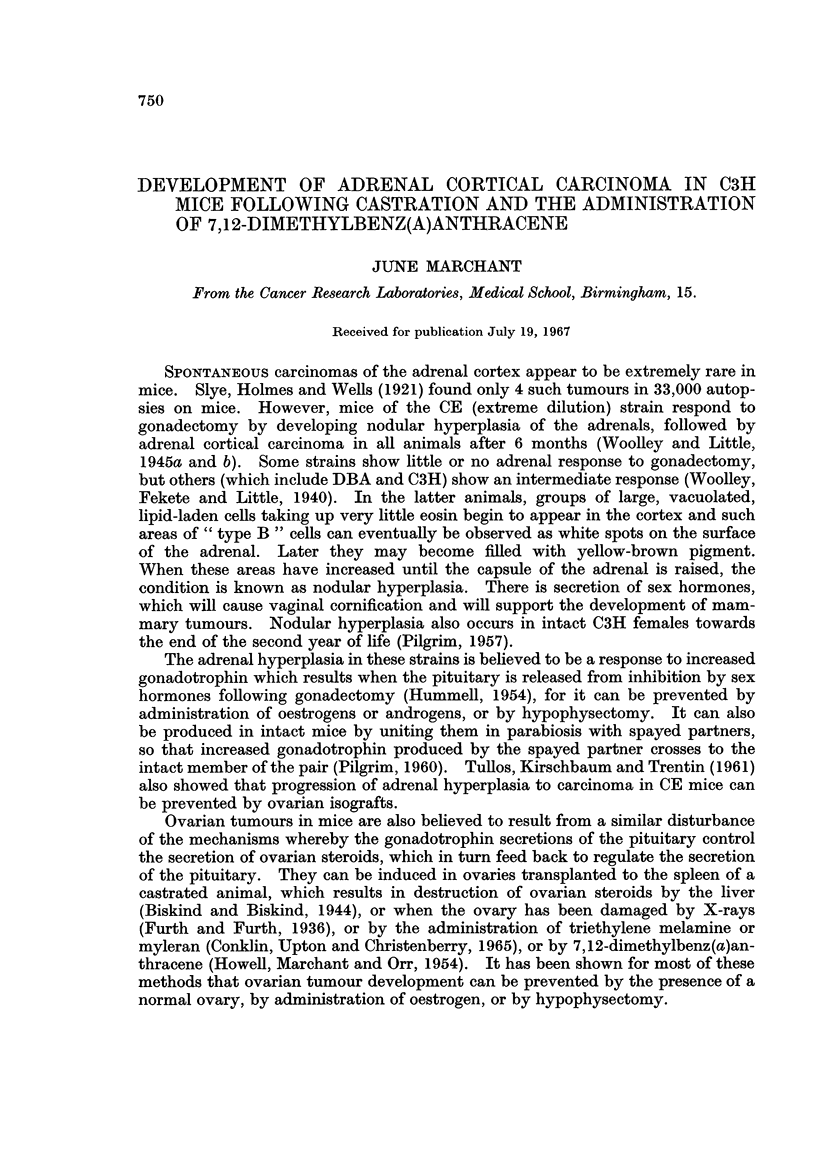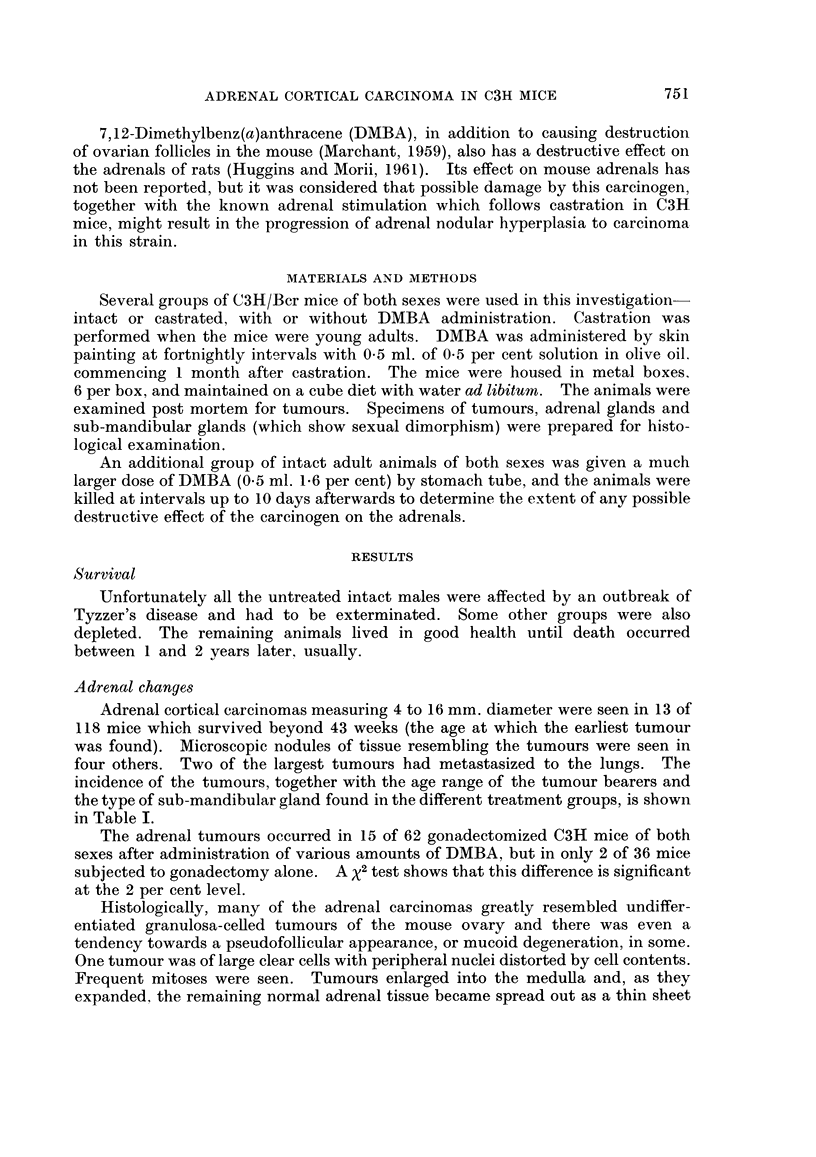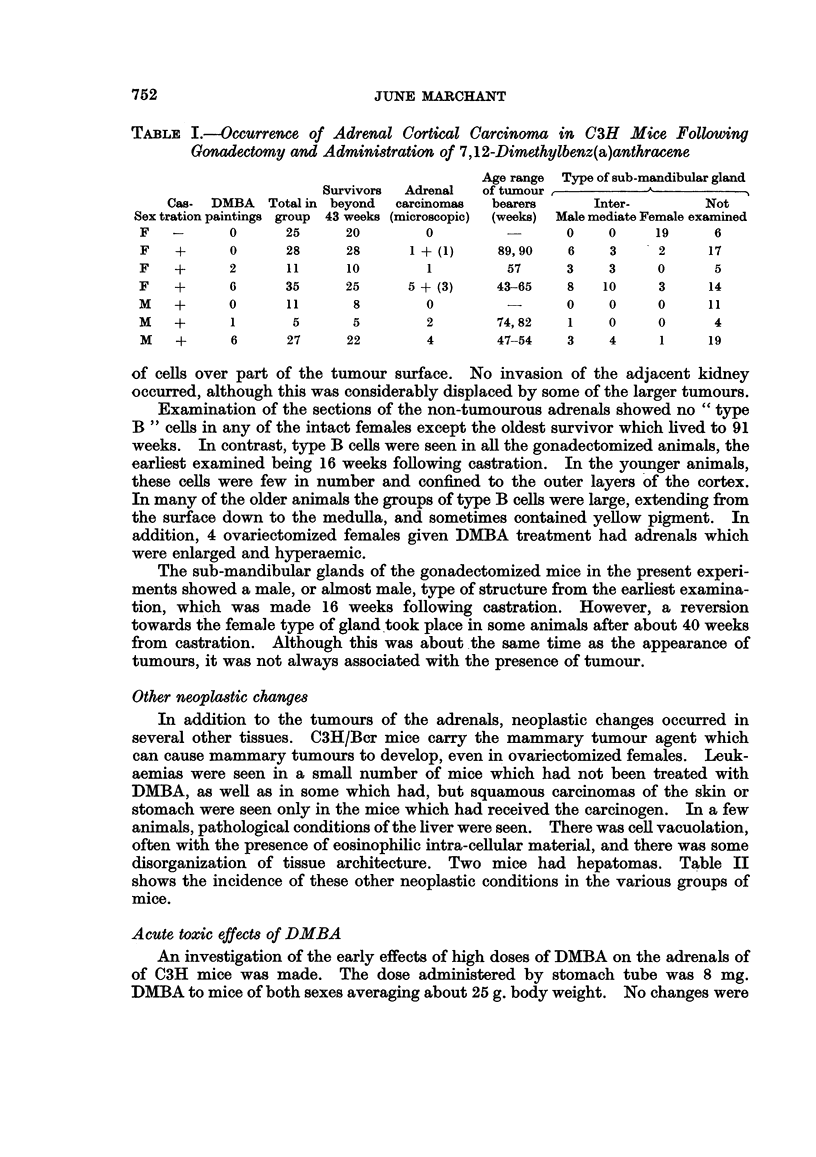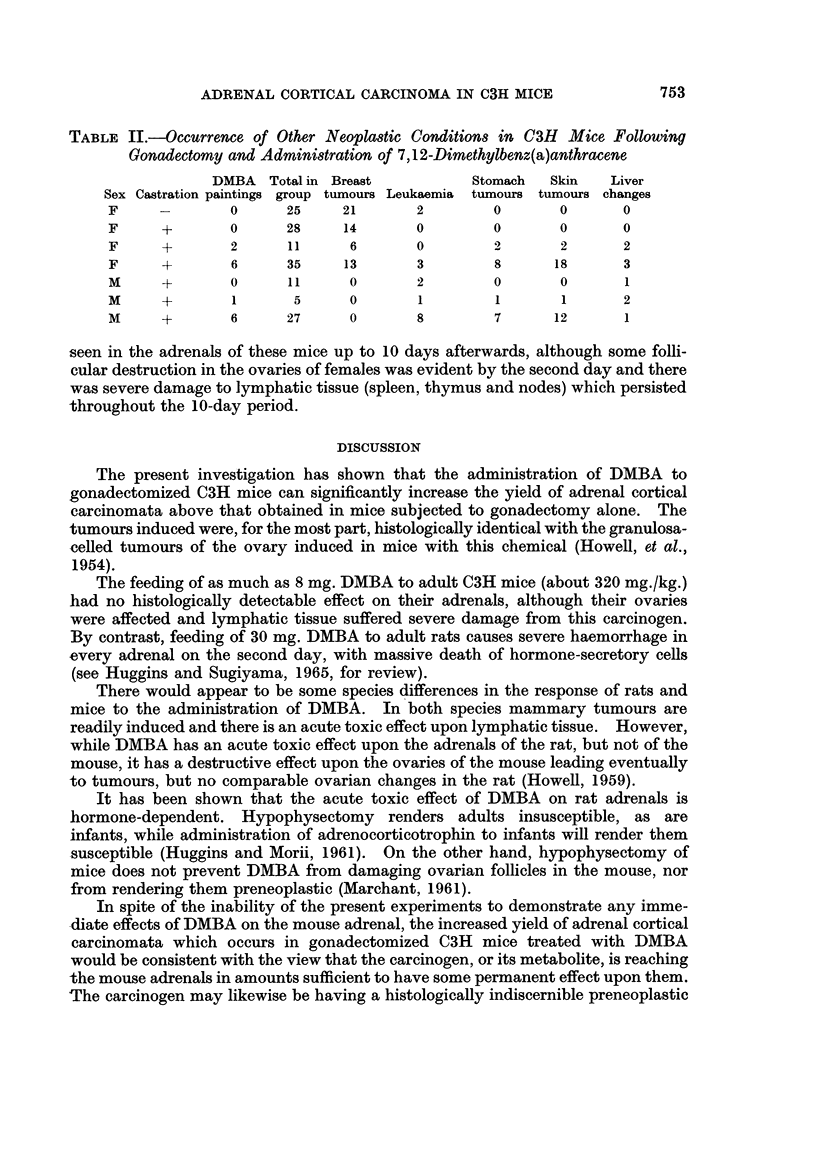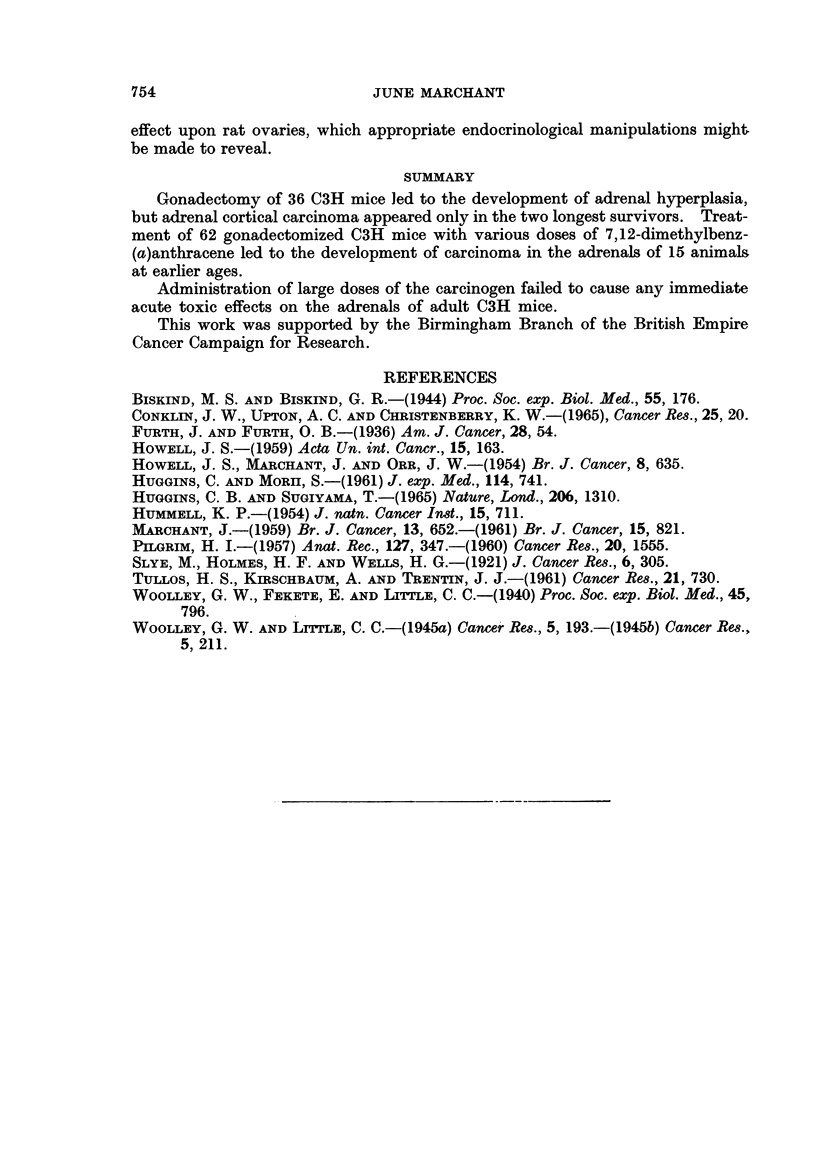# Development of adrenal cortical carcinoma in C3H mice following castration and the administration of 7,12-dimethylbenz(a)anthracene.

**DOI:** 10.1038/bjc.1967.87

**Published:** 1967-12

**Authors:** J. Marchant


### DEVELOPMENT OF ADRENAL CORTICAL CARCINOMA IN C3H MICE FOLLOWING CASTRATION AND THE ADMINISTRATION OF 7,12-DIMETHYLBENZ(A)ANTHRACENE

JUNE MARCHANT

*From the Cancer Research Laboratories, Medical School, Birmingham,* 15.

Received for publication July 19, 1967

SPONTANEOUS carcinomas of the adrenal cortex appear to be extremely rare in mice. Slye, Holmes and Wells (1921) found only 4 such tumours in 33,000 autopsies on mice. However, mice of the CE (extreme dilution) strain respond to gonadectomy by developing nodular hyperplasia of the adrenals, followed by adrenal cortical carcinoma in all animals after 6 months (Woolley and Little, 1945*a* and *b*). Some strains show little or no adrenal response to gonadectomy, but others (which include DBA and C3H) show an intermediate response (Woolley, Fekete and Little, 1940). In the latter animals, groups of large, vacuolated, lipid-laden cells taking up very little eosin begin to appear in the cortex and such areas of " type B " cells can eventually be observed as white spots on the surface of the adrenal. Later they may become filled with yellow-brown pigment. When these areas have increased until the capsule of the adrenal is raised, the condition is known as nodular hyperplasia. There is secretion of sex hormones, which will cause vaginal cornification and will support the development of mammary tumours. Nodular hyperplasia also occurs in intact C3H females towards the end of the second year of life (Pilgrim, 1957).

The adrenal hyperplasia in these strains is believed to be a response to increased gonadotrophin which results when the pituitary is released from inhibition by sex hormones following gonadectomy (Hummell, 1954), for it can be prevented by administration of oestrogens or androgens, or by hypophysectomy. It can also be produced in intact mice by uniting them in parabiosis with spayed partners, so that increased gonadotrophin produced by the spayed partner crosses to the intact member of the pair (Pilgrim, 1960). Tullos, Kirschbaum and Trentin (1961) also showed that progression of adrenal hyperplasia to carcinoma in CE mice can be prevented by ovarian isografts.

Ovarian tumours in mice are also believed to result from a similar disturbance of the mechanisms whereby the gonadotrophin secretions of the pituitary control the secretion of ovarian steroids, which in turn feed back to regulate the secretion of the pituitary. They can be induced in ovaries transplanted to the spleen of a castrated animal, which results in destruction of ovarian steroids by the liver (Biskind and Biskind, 1944), or when the ovary has been damaged by X-rays (Furth and Furth, 1936), or by the administration of triethylene melamine or myleran (Conklin, Upton and Christenberry, 1965), or by 7,12-dimethylbenz(*a*)anthracene (Howell, Marchant and Orr, 1954). It has been shown for most of these methods that ovarian tumour development can be prevented by the presence of a normal ovary, by administration of oestrogen, or by hypophysectomy.

7,12-Dimethylbenz(*a*)anthracene (DMBA), in addition to causing destruction of ovarian follicles in the mouse (Marchant, 1959), also has a destructive effect on the adrenals of rats (Huggins and Morii, 1961). Its effect on mouse adrenals has not been reported, but it was considered that possible damage by this carcinogen, together with the known adrenal stimulation which follows castration in C3H mice, might result in the progression of adrenal nodular hyperplasia to carcinoma in this strain.

### MATERIALS AND METHODS

Several groups of C3H/Bcr mice of both sexes were used in this investigation—intact or castrated, with or without DMBA administration. Castration was performed when the mice were young adults. DMBA was administered by skin painting at fortnightly intervals with 0·5 ml. of 0·5 per cent solution in olive oil. commencing 1 month after castration. The mice were housed in metal boxes. 6 per box, and maintained on a cube diet with water *ad libitum*. The animals were examined post mortem for tumours. Specimens of tumours, adrenal glands and sub-mandibular glands (which show sexual dimorphism) were prepared for histological examination.

An additional group of intact adult animals of both sexes was given a much larger dose of DMBA (0·5 ml. 1·6 per cent) by stomach tube, and the animals were killed at intervals up to 10 days afterwards to determine the extent of any possible destructive effect of the carcinogen on the adrenals.

### RESULTS

### *Survival*

Unfortunately all the untreated intact males were affected by an outbreak of Tyzzer's disease and had to be exterminated. Some other groups were also depleted. The remaining animals lived in good health until death occurred between 1 and 2 years later, usually.

### *Adrenal changes*

Adrenal cortical carcinomas measuring 4 to 16 mm. diameter were seen in 13 of 118 mice which survived beyond 43 weeks (the age at which the earliest tumour was found). Microscopic nodules of tissue resembling the tumours were seen in four others. Two of the largest tumours had metastasized to the lungs. The incidence of the tumours, together with the age range of the tumour bearers and the type of sub-mandibular gland found in the different treatment groups, is shown in Table I.

The adrenal tumours occurred in 15 of 62 gonadectomized C3H mice of both sexes after administration of various amounts of DMBA, but in only 2 of 36 mice subjected to gonadectomy alone. A $\chi^2$ test shows that this difference is significant at the 2 per cent level.

Histologically, many of the adrenal carcinomas greatly resembled undifferentiated granulosa-celled tumours of the mouse ovary and there was even a tendency towards a pseudofollicular appearance, or mucoid degeneration, in some. One tumour was of large clear cells with peripheral nuclei distorted by cell contents. Frequent mitoses were seen. Tumours enlarged into the medulla and, as they expanded, the remaining normal adrenal tissue became spread out as a thin sheet

TABLE I.—*Occurrence of Adrenal Cortical Carcinoma in C3H Mice Following Gonadectomy and Administration of* 7,12-*Dimethylbenz*(a)*anthracene*

| Sex | Cas-tration | DMBA paintings | Total in group | Survivors beyond 43 weeks | Adrenal carcinomas (microscopic) | Age range of tumour bearers (weeks) | Type of sub-mandibular gland | | | |
|---|---|---|---|---|---|---|---|---|---|---|
| | | | | | | | Male | Inter-mediate | Female | Not examined |
| F | — | 0 | 25 | 20 | 0 | — | 0 | 0 | 19 | 6 |
| F | + | 0 | 28 | 28 | 1 + (1) | 89, 90 | 6 | 3 | 2 | 17 |
| F | + | 2 | 11 | 10 | 1 | 57 | 3 | 3 | 0 | 5 |
| F | + | 6 | 35 | 25 | 5 + (3) | 43–65 | 8 | 10 | 3 | 14 |
| M | + | 0 | 11 | 8 | 0 | — | 0 | 0 | 0 | 11 |
| M | + | 1 | 5 | 5 | 2 | 74, 82 | 1 | 0 | 0 | 4 |
| M | + | 6 | 27 | 22 | 4 | 47–54 | 3 | 4 | 1 | 19 |

of cells over part of the tumour surface. No invasion of the adjacent kidney occurred, although this was considerably displaced by some of the larger tumours.

Examination of the sections of the non-tumourous adrenals showed no " type B " cells in any of the intact females except the oldest survivor which lived to 91 weeks. In contrast, type B cells were seen in all the gonadectomized animals, the earliest examined being 16 weeks following castration. In the younger animals, these cells were few in number and confined to the outer layers of the cortex. In many of the older animals the groups of type B cells were large, extending from the surface down to the medulla, and sometimes contained yellow pigment. In addition, 4 ovariectomized females given DMBA treatment had adrenals which were enlarged and hyperaemic.

The sub-mandibular glands of the gonadectomized mice in the present experiments showed a male, or almost male, type of structure from the earliest examination, which was made 16 weeks following castration. However, a reversion towards the female type of gland took place in some animals after about 40 weeks from castration. Although this was about the same time as the appearance of tumours, it was not always associated with the presence of tumour.

*Other neoplastic changes*

In addition to the tumours of the adrenals, neoplastic changes occurred in several other tissues. C3H/Bcr mice carry the mammary tumour agent which can cause mammary tumours to develop, even in ovariectomized females. Leukaemias were seen in a small number of mice which had not been treated with DMBA, as well as in some which had, but squamous carcinomas of the skin or stomach were seen only in the mice which had received the carcinogen. In a few animals, pathological conditions of the liver were seen. There was cell vacuolation, often with the presence of eosinophilic intra-cellular material, and there was some disorganization of tissue architecture. Two mice had hepatomas. Table II shows the incidence of these other neoplastic conditions in the various groups of mice.

*Acute toxic effects of DMBA*

An investigation of the early effects of high doses of DMBA on the adrenals of of C3H mice was made. The dose administered by stomach tube was 8 mg. DMBA to mice of both sexes averaging about 25 g. body weight. No changes were

TABLE II.—*Occurrence of Other Neoplastic Conditions in C3H Mice Following Gonadectomy and Administration of* 7,12-*Dimethylbenz*(a)*anthracene*

| Sex | Castration | DMBA paintings | Total in group | Breast tumours | Leukaemia | Stomach tumours | Skin tumours | Liver changes |
|---|---|---|---|---|---|---|---|---|
| F | – | 0 | 25 | 21 | 2 | 0 | 0 | 0 |
| F | + | 0 | 28 | 14 | 0 | 0 | 0 | 0 |
| F | + | 2 | 11 | 6 | 0 | 2 | 2 | 2 |
| F | + | 6 | 35 | 13 | 3 | 8 | 18 | 3 |
| M | + | 0 | 11 | 0 | 2 | 0 | 0 | 1 |
| M | + | 1 | 5 | 0 | 1 | 1 | 1 | 2 |
| M | + | 6 | 27 | 0 | 8 | 7 | 12 | 1 |

seen in the adrenals of these mice up to 10 days afterwards, although some follicular destruction in the ovaries of females was evident by the second day and there was severe damage to lymphatic tissue (spleen, thymus and nodes) which persisted throughout the 10-day period.

### DISCUSSION

The present investigation has shown that the administration of DMBA to gonadectomized C3H mice can significantly increase the yield of adrenal cortical carcinomata above that obtained in mice subjected to gonadectomy alone. The tumours induced were, for the most part, histologically identical with the granulosa-celled tumours of the ovary induced in mice with this chemical (Howell, *et al.*, 1954).

The feeding of as much as 8 mg. DMBA to adult C3H mice (about 320 mg./kg.) had no histologically detectable effect on their adrenals, although their ovaries were affected and lymphatic tissue suffered severe damage from this carcinogen. By contrast, feeding of 30 mg. DMBA to adult rats causes severe haemorrhage in every adrenal on the second day, with massive death of hormone-secretory cells (see Huggins and Sugiyama, 1965, for review).

There would appear to be some species differences in the response of rats and mice to the administration of DMBA. In both species mammary tumours are readily induced and there is an acute toxic effect upon lymphatic tissue. However, while DMBA has an acute toxic effect upon the adrenals of the rat, but not of the mouse, it has a destructive effect upon the ovaries of the mouse leading eventually to tumours, but no comparable ovarian changes in the rat (Howell, 1959).

It has been shown that the acute toxic effect of DMBA on rat adrenals is hormone-dependent. Hypophysectomy renders adults insusceptible, as are infants, while administration of adrenocorticotrophin to infants will render them susceptible (Huggins and Morii, 1961). On the other hand, hypophysectomy of mice does not prevent DMBA from damaging ovarian follicles in the mouse, nor from rendering them preneoplastic (Marchant, 1961).

In spite of the inability of the present experiments to demonstrate any immediate effects of DMBA on the mouse adrenal, the increased yield of adrenal cortical carcinomata which occurs in gonadectomized C3H mice treated with DMBA would be consistent with the view that the carcinogen, or its metabolite, is reaching the mouse adrenals in amounts sufficient to have some permanent effect upon them. The carcinogen may likewise be having a histologically indiscernible preneoplastic effect upon rat ovaries, which appropriate endocrinological manipulations might be made to reveal.

### SUMMARY

Gonadectomy of 36 C3H mice led to the development of adrenal hyperplasia, but adrenal cortical carcinoma appeared only in the two longest survivors. Treatment of 62 gonadectomized C3H mice with various doses of 7,12-dimethylbenz(*a*)anthracene led to the development of carcinoma in the adrenals of 15 animals at earlier ages.

Administration of large doses of the carcinogen failed to cause any immediate acute toxic effects on the adrenals of adult C3H mice.

This work was supported by the Birmingham Branch of the British Empire Cancer Campaign for Research.

### REFERENCES

Biskind, M. S. and Biskind, G. R.—(1944) *Proc. Soc. exp. Biol. Med.*, **55**, 176.
Conklin, J. W., Upton, A. C. and Christenberry, K. W.—(1965), *Cancer Res.*, **25**, 20.
Furth, J. and Furth, O. B.—(1936) *Am. J. Cancer*, **28**, 54.
Howell, J. S.—(1959) *Acta Un. int. Cancr.*, **15**, 163.
Howell, J. S., Marchant, J. and Orr, J. W.—(1954) *Br. J. Cancer*, **8**, 635.
Huggins, C. and Morii, S.—(1961) *J. exp. Med.*, **114**, 741.
Huggins, C. B. and Sugiyama, T.—(1965) *Nature, Lond.*, **206**, 1310.
Hummell, K. P.—(1954) *J. natn. Cancer Inst.*, **15**, 711.
Marchant, J.—(1959) *Br. J. Cancer*, **13**, 652.—(1961) *Br. J. Cancer*, **15**, 821.
Pilgrim, H. I.—(1957) *Anat. Rec.*, **127**, 347.—(1960) *Cancer Res.*, **20**, 1555.
Slye, M., Holmes, H. F. and Wells, H. G.—(1921) *J. Cancer Res.*, **6**, 305.
Tullos, H. S., Kirschbaum, A. and Trentin, J. J.—(1961) *Cancer Res.*, **21**, 730.
Woolley, G. W., Fekete, E. and Little, C. C.—(1940) *Proc. Soc. exp. Biol. Med.*, **45**, 796.
Woolley, G. W. and Little, C. C.—(1945*a*) *Cancer Res.*, **5**, 193.—(1945*b*) *Cancer Res.*, **5**, 211.